# Maize root mucilage alters stomatal responses to soil and atmospheric drought: Implications for plant water use

**DOI:** 10.1093/plphys/kiaf510

**Published:** 2025-10-13

**Authors:** Gaochao Cai, Asegidew Akale, Efstathios Diamantopoulos, Frederic Leuther, Lara Kersting, Scott McAdam, Shurong Liu, Mutez A Ahmed

**Affiliations:** School of Agriculture and Biotechnology, Shenzhen Campus of Sun Yat-sen University, 518107, Shenzhen, China; Root-Soil Interaction, School of Life Sciences, Technical University of Munich, 85354, Freising, Germany; Chair of Soil Physics, University of Bayreuth, 95447, Bayreuth, Germany; Chair of Soil Physics, University of Bayreuth, 95447, Bayreuth, Germany; Chair of Soil Physics, University of Bayreuth, 95447, Bayreuth, Germany; Department of Botany and Plant Pathology, Purdue University, West Lafayette, IN 47907, USA; School of Agriculture and Biotechnology, Shenzhen Campus of Sun Yat-sen University, 518107, Shenzhen, China; Root-Soil Interaction, School of Life Sciences, Technical University of Munich, 85354, Freising, Germany

## Abstract

Plants respond to soil and atmospheric water deficits through strategies such as stomatal regulation and belowground adaptations. Root mucilage buffers erratic fluctuations in the rhizosphere water content, yet its influence on soil hydraulic properties, especially unsaturated hydraulic conductivity, and stomatal regulation remains unknown. We hypothesized that mucilage facilitates water uptake by attenuating the drop in matric potential at the root–soil interface during soil and atmospheric drying. We measured the impact of various maize (*Zea mays*) mucilage contents (0.0%, 0.05%, 0.2%, and 0.4%) on the water retention and hydraulic conductivity of a loamy soil. Leveraging a soil–plant hydraulic model, we investigated the effects of mucilage contents on transpiration and stomatal responses under soil drying and increased vapor pressure deficit (VPD). Higher mucilage contents prevented sharp declines in unsaturated hydraulic conductivity as soils dried. Simulations revealed that higher mucilage contents delayed the onset of hydraulic stress (the threshold transpiration rate beyond which a small increase in transpiration would result in a disproportionate decline in leaf water potential), broadened the hydroscape zone, and shifted stomatal behavior from isohydric to more anisohydric regulation, enabling plants to sustain stable transpiration and lower midday leaf water potentials under drought. The buffering effects on soil–plant hydraulics persisted across varying degrees of VPD, although high mucilage contents accelerated soil drying, indicating a trade-off between improved water uptake and faster moisture depletion during prolonged drought. Our findings underscore the important role of mucilage in modulating soil–plant water relations and stomatal regulation, offering insights into strategies for improving plant responses to soil and atmospheric drought.

## Teaser

Increased maize root mucilage enhances unsaturated hydraulic conductivity, delays hydraulic stress onset, and shifts toward anisohydric stomatal behavior under drought but accelerates soil drying.

## Introduction

Plants experience water stress when soil water availability is limited, when atmospheric water demand is elevated, or when a combination of these 2 factors occurs. Water movement from soil to plants depends on the gradients in water potential (Tyree 1997; Mencuccini et al. 2019) and is influenced by the hydraulic properties of both soil and roots (Tardieu et al. 2018; Cai et al. 2022). In well-hydrated soil, root resistance often limits water transport, while the impact of soil hydraulic conductivity remains negligible. As soil dries, soil resistance increases by 2 to 4 orders of magnitude and leads to a large drop in soil–plant hydraulic conductance, limiting root water uptake (Passioura 1982; Draye et al. 2010). Recently, this decrease in below-ground hydraulic conductance has been identified as the main limiting factor that triggers stomatal closure (Rodriguez-Dominguez and Brodribb 2020; Bourbia et al. 2021; Abdalla et al. 2022; Manandhar et al. 2024). These findings emphasize the putative role of the soil–root interface in controlling root water uptake and plant water use (Ahmed et al. 2018a).

To enhance water uptake under edaphic stress, plants have evolved numerous adaptations, including the development of root hairs (Cai and Ahmed 2022), activation of aquaporins (Ache et al. 2010; Maurel et al. 2010; Lobet et al. 2014), hydropatterning (Bao et al. 2014), suberization of the root tissue (Cantó-Pastor et al. 2024), associations with arbuscular mycorrhizal fungi (Abdalla et al. 2023), and the exudation of mucilage (Carminati et al. 2010; Ahmed et al. 2014; Abdalla et al. 2024). Mucilage, a gelatinous viscoelastic substance secreted by root-cap border cells in most vascular plants—including cereals, legumes, and many dicots (Cai et al. 2013; Jiang et al. 2023), plays a crucial role in modifying the hydraulic properties of the rhizosphere (Czarnes et al. 2000; Carminati et al. 2017; Ahmed et al. 2018a). Composed primarily of polysaccharides with smaller proportions of proteins, minerals, and lipids (Naveed et al. 2017; Nazari et al. 2020), mucilage creates a polymer network that interacts with the soil matrix to affect soil hydraulic properties, with effects that vary depending on the species, developmental stage, and environmental conditions (Nazari et al. 2020; Werner et al. 2022; Santangeli et al. 2024; Knott et al. 2022). Despite its importance, the interactions between mucilage, soil hydraulic properties, and plant physiological responses to water stress remain poorly understood.

Mucilage has been proposed to improve water fluxes by increasing water availability and maintaining water flow to roots during soil drying (Carminati 2012; Carminati et al. 2017; Xiao et al. 2024; Abdalla et al. 2024). Kroener et al. (2018) observed that chia (*Salvia hispanica*) seed mucilage increases soil water retention in the rhizosphere. Acting as a critical interface between roots and the surrounding soil, mucilage has been hypothesized to modify the water potential range over which roots and soil remain hydraulically connected (Carminati et al. 2011; Ahmed et al. 2014). This effect arises from its ability to reduce the surface tension, increase the viscosity of the soil solution, and maintain the liquid-phase connectivity during soil drying (Carminati et al. 2017; Werner et al. 2022; Diehl et al. 2023). Due to its high viscosity, mucilage significantly decreases the saturated soil hydraulic conductivity by several orders of magnitude (Kroener et al. 2014; Benard et al. 2019; Volk et al. 2016; Ahmed et al. 2018c), which could impose mild water stress on the plant, leading to a reduction in the transpiration rate (Berauer et al. 2023; Ahmed et al. 2018c). Simulation studies by Landl et al. (2021) and Schwartz et al. (2016) have proposed that the presence of mucilage in the rhizosphere prevents fast dehydration of the root by decreasing transpiration during soil drying. As a globally grown staple crop with relatively high mucilage exudation, maize (*Zea mays*) provides an ideal system to investigate these effects. Despite its importance, the impact of maize root mucilage and its varying contents on soil hydraulic properties remains unclear. In particular, the impact of root mucilage on unsaturated hydraulic conductivity has received little attention, largely because most studies used seed-derived mucilage or artificial media, and hence the downstream effects on plant water use under both soil and atmospheric drying are yet to be fully characterized.

Understanding the role of root mucilage in plant water uptake under both soil and atmospheric drying conditions poses several challenges. Numerous previous studies have primarily examined mucilage effects on soil or rhizosphere hydraulic properties using seed-extracted gels (e.g. chia; Kroener et al. 2018; Naveed et al. 2019), or by combining these seed-derived gels with artificial substrates such as glass beads (Benard et al. 2018). Furthermore, prior studies have focused on the physical properties of mucilage (Werner et al. 2022; Berauer et al. 2023; Knott et al. 2022) and its immediate effects on the soil environment, with limited attention paid to plant physiological responses, such as stomatal regulation, which are ultimately influenced by these changes (Schwartz et al. 2016; Landl et al. 2021). To our knowledge, only a few studies, if any, have specifically investigated mucilage directly collected from crop roots, particularly maize roots grown under field conditions, to explore its effects on soil hydraulic properties, especially the unsaturated soil hydraulic conductivity and root water uptake. Additionally, practical challenges exist in establishing reproducible experimental conditions when working with maize genotypes that vary in mucilage production, as such genotypes are not widely available. Addressing these limitations, our study collected mucilage directly from maize roots grown in the field to examine the effects on soil hydraulic properties in natural soils. Furthermore, we investigated how varying mucilage contents influence soil hydraulic properties and, subsequently, plant water uptake responses under both soil and atmospheric drying conditions. Specifically, these responses were assessed under different vapor pressure deficit (VPD) levels to simulate varying transpirational demands.

**Figure 1.**
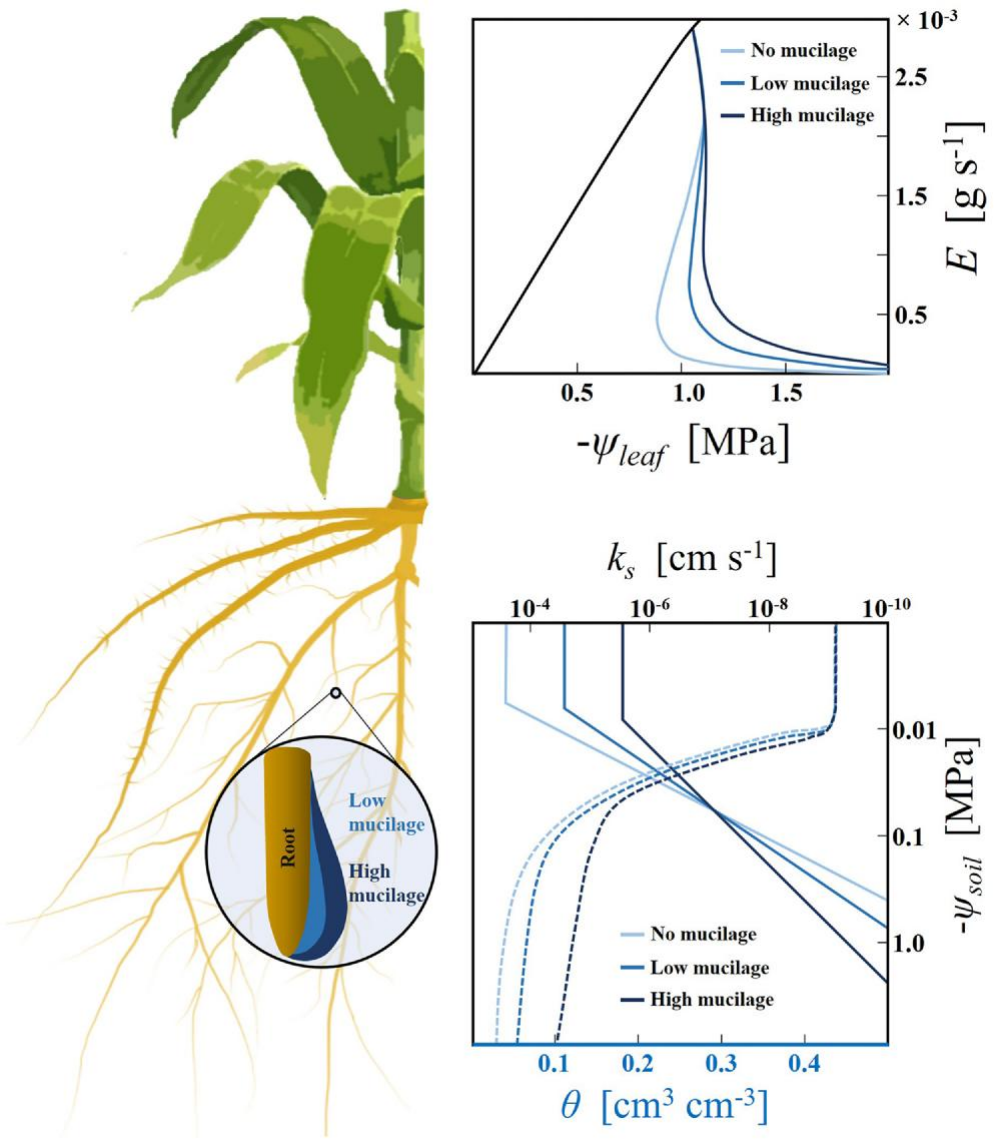
Hypothetical effects of root mucilage content on soil hydraulic properties and the relationship between leaf water potential (ψ_leaf_) and transpiration rate (*E*). High mucilage content (dark blue line) elevates soil water content (*θ*) and reduces saturated soil hydraulic conductivity (bottom right). Solid and dashed lines are soil water retention and hydraulic conductivity curves, respectively. However, it also slows down the decline in unsaturated soil hydraulic conductivity (*k*_s_) due to increased viscosity and reduced surface tension of soil water. These effects mitigate the decline in matric potential at the soil–root interface and enable plants to maintain higher transpiration rates at lower soil matric potential, thus postponing the SOL (blue lines) (top right). SOL is defined as the point at which the slope of the transpiration rate (*E*) versus leaf water potential (ψ_leaf_) curve declines to 60% to 80% of its maximum value, thereby representing the maximum transpiration rate that can be sustained before the risk of hydraulic failure increases significantly. Only the isoline of the *E*(ψ_leaf_) relationship (black line) at the highest soil matric potential is shown for a clear illustration.

Moreover, the interaction between varying mucilage contents and soil drying on water uptake and stomatal regulation remains insufficiently explored. This modeling study represents a step toward addressing these knowledge gaps by (i) quantifying the impact of varying maize mucilage contents on soil hydraulic properties, and (ii) leveraging a soil–plant hydraulic model to investigate how different maize mucilage contents may influence root water uptake and stomatal regulation under varying soil and atmospheric drying conditions. We hypothesize that mucilage increases soil water content at a given water potential, with the effect increasing with mucilage content (Fig. 1). Under near saturated conditions, high mucilage contents are expected to reduce the saturated hydraulic conductivity by 2 to 3 orders of magnitude compared to soils with low or no mucilage (Fig. 1). As soils dry, higher mucilage contents are hypothesized to enhance water retention and mitigate a decline in soil hydraulic conductivity, thereby slowing down the decline in soil–plant hydraulic conductance, sustaining higher transpiration rates at more negative leaf water potentials, and delaying the onset of water stress (Fig. 1). This is because mucilage forms a more continuous liquid phase and mitigates the drop in soil water potential during drying, thereby sustaining hydraulic connectivity and supporting water transport to plants. We acknowledge that these model predictions require experimental validation, and we present them here as a foundation for future empirical testing.

To test these hypotheses, we collected root mucilage from field-grown maize and measured soil hydraulic properties in soils mixed with 3 different mucilage contents using the evaporation method. Then we used a soil–plant hydraulic model to evaluate how the experimentally measured effects of varying mucilage contents on soil hydraulic properties influence root water uptake and transpiration under different atmospheric water demands and soil drying conditions.

## Results

### Soil water retention and hydraulic conductivity curves

**Figure 2.**
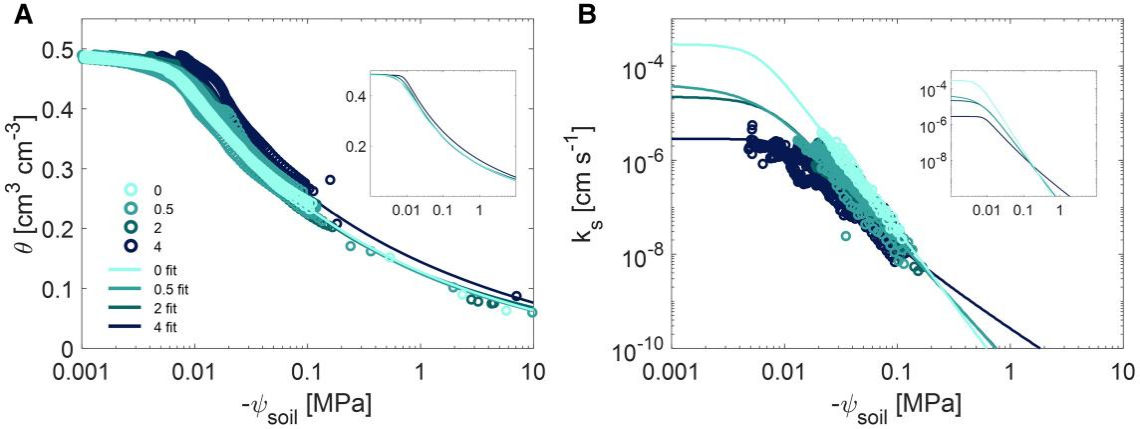
Soil water retention and hydraulic conductivity as a function of maize root mucilage content in a loamy soil. **A)** The measured and fitted soil water retention curves of the loamy soil mixed with 4 maize root mucilage contents. **B)** The measured and fitted soil hydraulic conductivity curves for 4 maize root mucilage contents. The sample sizes (*n*) were *n* = 69, 128, 148, and 273 (due to the distribution of measurement points) for mucilage contents of 0, 0.5, 2, and 4 mg g^−1^. θ, soil water content; ψ_soil_, soil matric potential; *k*_s_, soil hydraulic conductivity. Circle: measured values. Line: fitted curves (fit). The colors represent different mucilage treatments: 0 (without mucilage), 0.5, 2, and 4 mg (mucilage) g^−1^ (soil). The fitted curves without measurements are also shown in the inset figures.

Mucilage content in soil significantly changed both the soil water retention and hydraulic conductivity curves (Fig. 2 and Supplementary Fig. S1). The water content at saturation was also impacted by mucilage content (Fig. 2A). When mucilage content increased from 0.5 to 2 mg g^−1^, a slight increase in soil water content was observed between matric potentials of −0.005 and −0.05 MPa compared to the control, but this enhancement diminished under drier soil conditions (Fig. 2A). As the mucilage content increased to 4 mg g^−1^, the soil water content was consistently and significantly (Supplementary Fig. S1) higher than that of the control at a soil matric potential of −0.004 MPa.

Mucilage not only significantly reduced the model-derived saturated soil hydraulic conductivity (*k*_sat_) but also reshaped the decline in hydraulic conductivity (Fig. 2B and Supplementary Fig. S1). Relative to the control without mucilage, *k*_sat_ was 0.13, 0.07, and 0.01 for soils amended with 0.5, 2, and 4 mg g^−1^ mucilage, respectively, indicating that the reduction in *k*_sat_ was significant (Supplementary Fig. S2; Supplementary Text). *k*_sat_ estimations were derived from model fits based on unsaturated hydraulic conductivity measurements obtained during soil drying, but the magnitude of *k*_sat_ remained consistent for soils with the same mucilage content, regardless of the model used to fit *k*_sat_ (Supplementary Fig. S2). As these soils dried, the slope of the soil hydraulic conductivity (*k*_s_) became less steep at lower soil water potentials for all mucilage levels (Fig. 2B). The reduction in slope magnitude was most pronounced in the treatment with 4 mg g^−1^ mucilage. Furthermore, the *k*_s_ curves for soils with 0.5 and 2 mg g^−1^ mucilage intersected the control at −0.247 MPa, whereas the curves for 4 mg g^−1^ mucilage crossed the control earlier at around −0.15 MPa, reflecting both a delayed onset and a stronger attenuation of conductivity decline under the highest mucilage treatment.

### Effect of varying mucilage contents and VPD on the relationship between transpiration and leaf water potential

**Figure 3.**
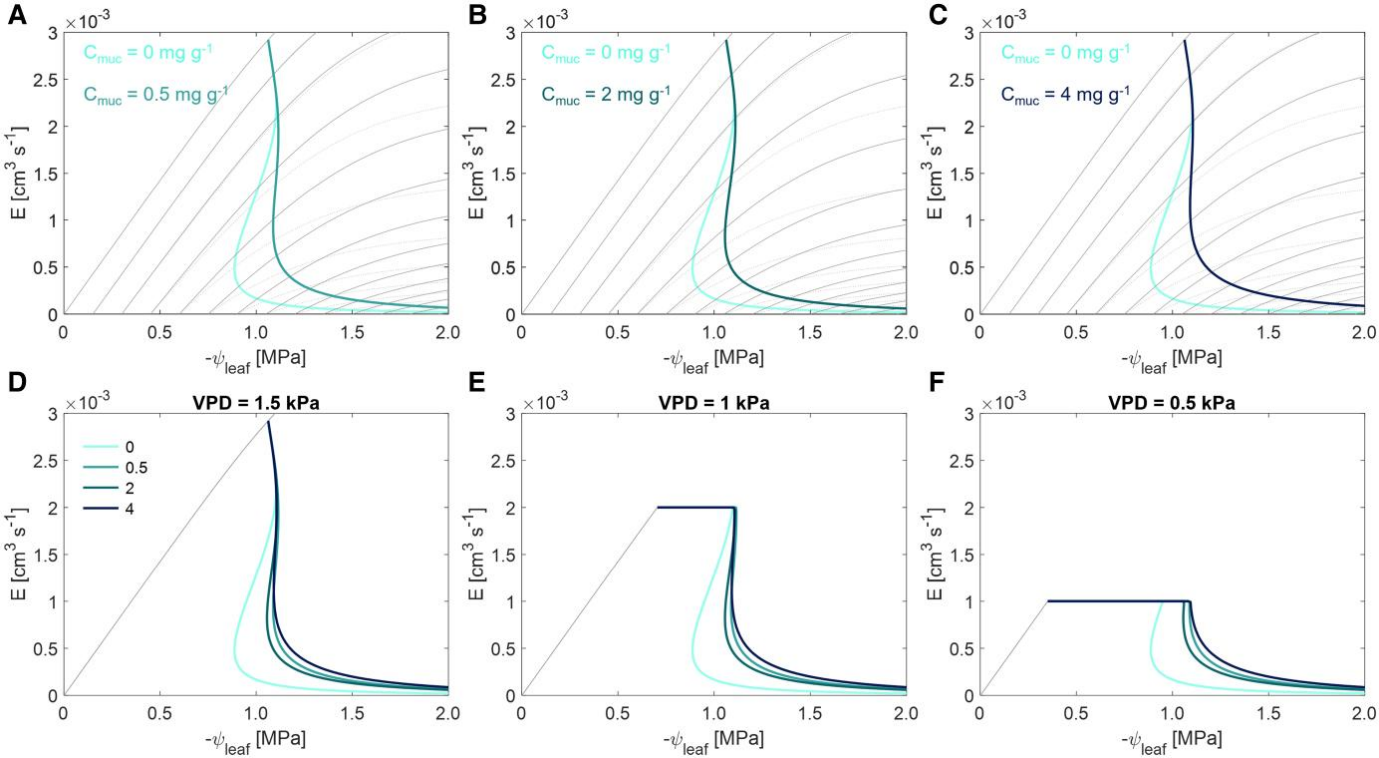
Transpiration response to soil drying with 4 mucilage contents under 3 different transpiration demand levels (VPD). The plots show the relationship between transpiration rate (*E*) and leaf water potential (ψ_leaf_) and the SOL curves (colored lines). **A)** to **C)** Comparison of the *E*(ψ_leaf_) relationship between soil with different mucilage contents (*C*_muc_) compared to the control (without mucilage). The dotted lines are the isolines of the *E*(ψ_leaf_) relationship from the control without mucilage, while the solid lines are from the mucilage treatments. **D)** to **F)** Comparison of the *E*(ψ_leaf_) relationship and corresponding SOL between soils with 4 mucilage contents (0, 0.5, 2, and 4 mg g^−1^) under 3 contrasting VPD levels (left to right, 1.5, 1, and 0.5 kPa). Only the isoline of the *E*(ψ_leaf_) relationship at the highest soil matric potential is shown for a clear illustration.

**Figure 4.**
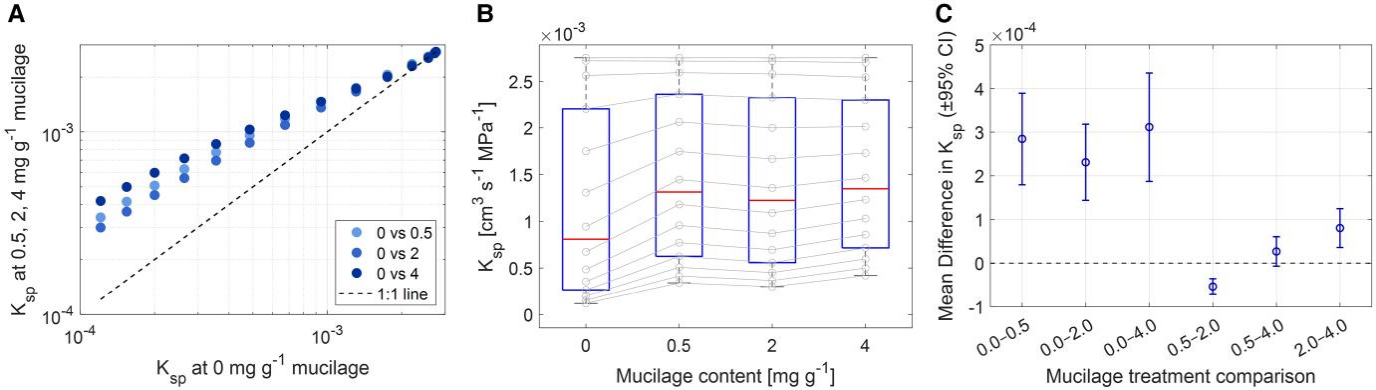
Comparison of soil–plant hydraulic conductance (*K*_sp_) across mucilage treatments (0, 0.5, 2, 4 mg g^−1^). **A)** Comparison of *K*_sp_ across mucilage treatments during soil drying. *K*_sp_ were calculated from Fig. 3A to C at soil matric potential of 0.001, 0.151, 0.301, 0.451, 0.601, 0.751, 0.901, 1.051, 1.201, 1.351, 1.501, 1.651, 1.801, and 1.951 MPa (points from right to left, and soil matric potential values were the intercepts on the *x*-axis of Fig. 3A to C). **B)** Distribution of *K*_sp_ for each mucilage treatment during soil drying (gray circles represent *K*_sp_ dynamics). Center line, median; box limits, upper and lower quartiles; whiskers, 1.5 times interquartile range; sample size *n* = 14. **C)** Pairwise comparisons of treatment means with 95% CI based on a 1-sample *t*-test of paired differences. Comparisons with CIs not overlapping zero indicate significant differences (*P* < 0.05).

By modifying the soil hydraulic properties, mucilage further modulates plant root water uptake and stomatal responses during soil drying. Regardless of the content, mucilage enabled plants to maintain a relatively higher transpiration rate at more negative leaf water potentials compared to the control, particularly as the soil dried (Fig. 3A and C). During soil drying, mucilage significantly mitigated the decline in soil–plant hydraulic conductance (*K*_sp_) and slope of the *E*(ψ_leaf_) relationship (Fig. 4). The effect was especially pronounced in plants with 4 mg g^−1^ mucilage content (Figs. 3D and 4A). The enhanced *K*_sp_ in turn facilitated water uptake and delayed the stress-onset limit (SOL) (Fig. 3). Interestingly, the SOL did not vary monotonically with mucilage content. For instance, plants with 2 mg g^−1^ mucilage content reached the stress limit earlier than those with 0.5 mg g^−1^ mucilage (Fig. 3D to F), which might be due to similar hydraulic conductivity curves (*K*_sat_ of the soil with 2 mg g^−1^ was half, and the decline slopes of the 2 soils were the same, also shown in Fig. 2B).

Although mucilage had a minor impact, if any, on the SOL with increasing VPD under wet soil conditions, it maintained the maximum transpiration rate under low VPD in drying soils. Under relatively low VPD levels (0.5 and 1 kPa), the SOL for different mucilage contents was rather similar to the ones under high VPD (1.5 kPa) (Fig. 3D to F), indicating mucilage continues to facilitate plant water uptake in drying soils regardless of VPD. Notably, under lower VPDs, the SOL was reached at more negative soil matric potentials: −0.311 MPa at medium VPD (1 kPa) and −0.591 MPa at the lowest VPD (0.5 kPa) (Fig. 3E and F).

### Matric potential at the soil–root interface with increasing transpiration rate under different VPD levels

**Figure 5.**
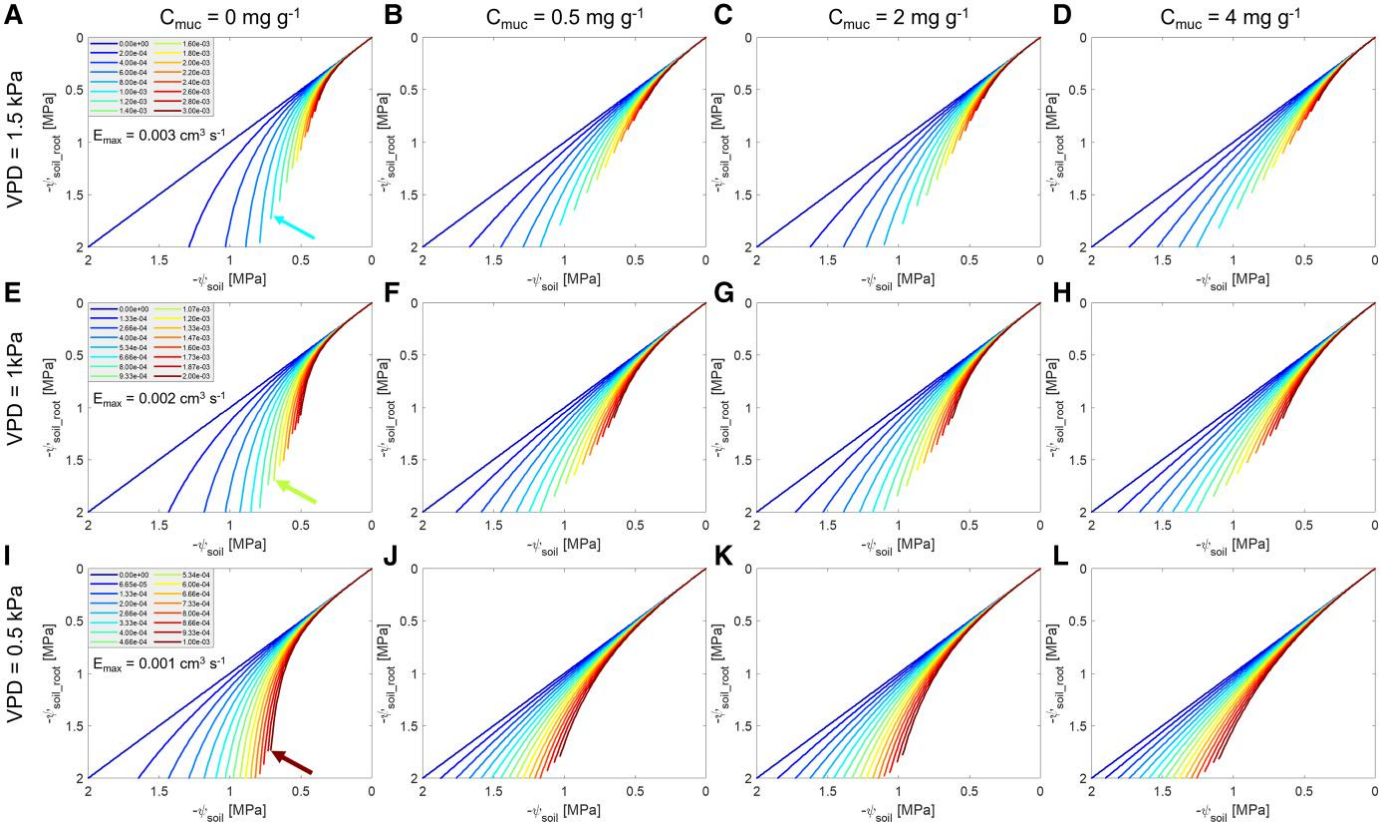
Variation in water potential at the soil–root interface (ψ_soil_root_) with increasing transpiration rate (*E*) in the loamy soil with 4 mucilage contents (*C*_muc_, left to right) and under 3 VPD conditions (top to bottom) during soil drying. Color from blue to red in each subplot presents increasing transpiration rate. The variations in water potential were shown with 16 selected transpiration rates ranging from the minimum to the maximum. The arrows in subplots **A)**, **E)**, and **I)** point to the lines at an identical transpiration rate under contrasting VPD conditions (see also Supplementary Fig. S3).

When plants transpire, the water potential at the soil–root interface (ψ_soil_root_) declines and diverges from the bulk soil matric potential. In dry soils, this deviation became larger with increasing transpiration rate and occurred at a less negative soil matric potential (Fig. 5). The deviation between ψ_soil_ and ψ_soil_root_ for varying transpiration rates was alleviated with increasing mucilage contents. For instance, at the lower transpiration rate of 2 × 10^−4^ cm^3^ s^−1^ (the second blue curve in Fig. 5A) and 4 × 10^−4^ cm^3^ s^−1^ (the third blue curve in Fig. 5A), the difference between ψ_soil_ and ψ_soil_root_ decreased with increasing mucilage content. Without mucilage, these differences were −0.71 and −0.97 MPa, respectively. With 0.5 mg g^−1^ mucilage, the differences were reduced to −0.33 and −0.55 MPa, and with 4 mg g^−1^ mucilage, they further decreased to −0.27 and −0.47 MPa. Reduced VPD resulted in lower maximum transpiration rates but did not change the relationship between ψ_soil_ and ψ_soil_root_ at equivalent transpiration rates and mucilage contents (the arrows in Fig. 5A, E, and I; see also Supplementary Fig. S3).

### Effect of varying mucilage contents on the hydroscape zone and the degree of isohydricity under different VPD levels

**Figure 6.**
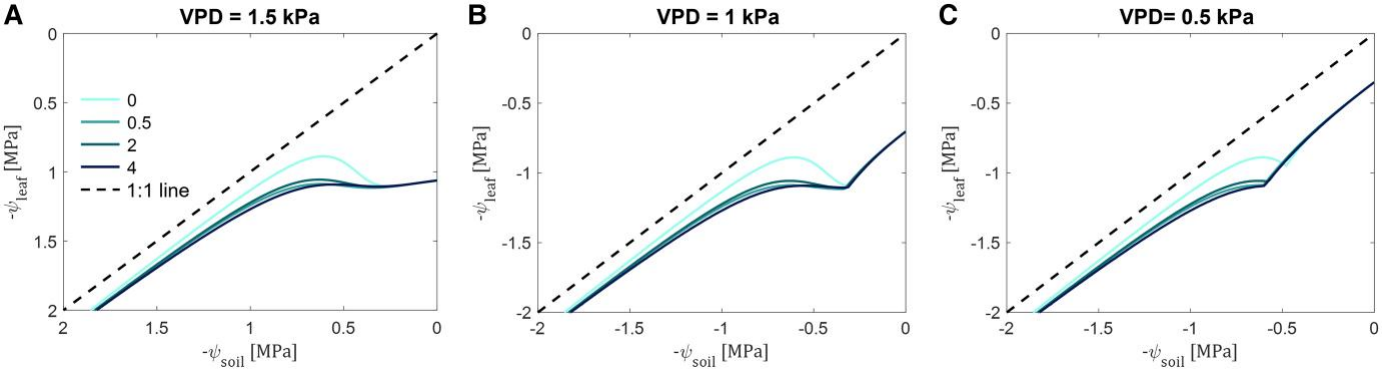
The relationships between leaf (ψ_leaf_) and soil (ψ_soil_) water potential in the loamy soil with 4 mucilage contents and under 3 VPD conditions during soil drying. The colors represent different mucilage treatments: 0 (without mucilage), 0.5, 2, and 4 mg (mucilage) g^−1^ (soil).

The relationship between daily minimum leaf water potential (ψ_leaf_MD_) and the corresponding soil matric potential (ψ_soil_), as captured by the regression line in Fig. 6 defines a hydroscape zone. This zone quantifies the soil–plant water potential range that governs stomatal regulation and a plant degree of isohydricity. Simulation results showed that higher mucilage content allowed ψ_leaf_MD_ to increasingly decline before plants start to close stomata, particularly when soil matric potential was lower than −0.49 MPa, resulting in broader hydroscape zones (Fig. 6A). This enlargement diminished as the soil continued to dry. The larger difference between soil and leaf water potential suggests a shift in stomatal regulation from isohydric to a more anisohydric behavior. Additionally, decreasing VPD resulted in a compression of the hydroscape zone across all mucilage contents (Fig. 6B and C). Specifically, at soil matric potentials between −0.4 and −0.5 MPa, the lower VPD level produced smaller differences between ψ_leaf_MD_ and soil matric potential. Despite this overall compression, increasing mucilage content still expanded the hydroscape zone at soil matric potentials below −0.49 MPa.

### Effect of mucilage content on transpiration during soil drying

**Figure 7.**
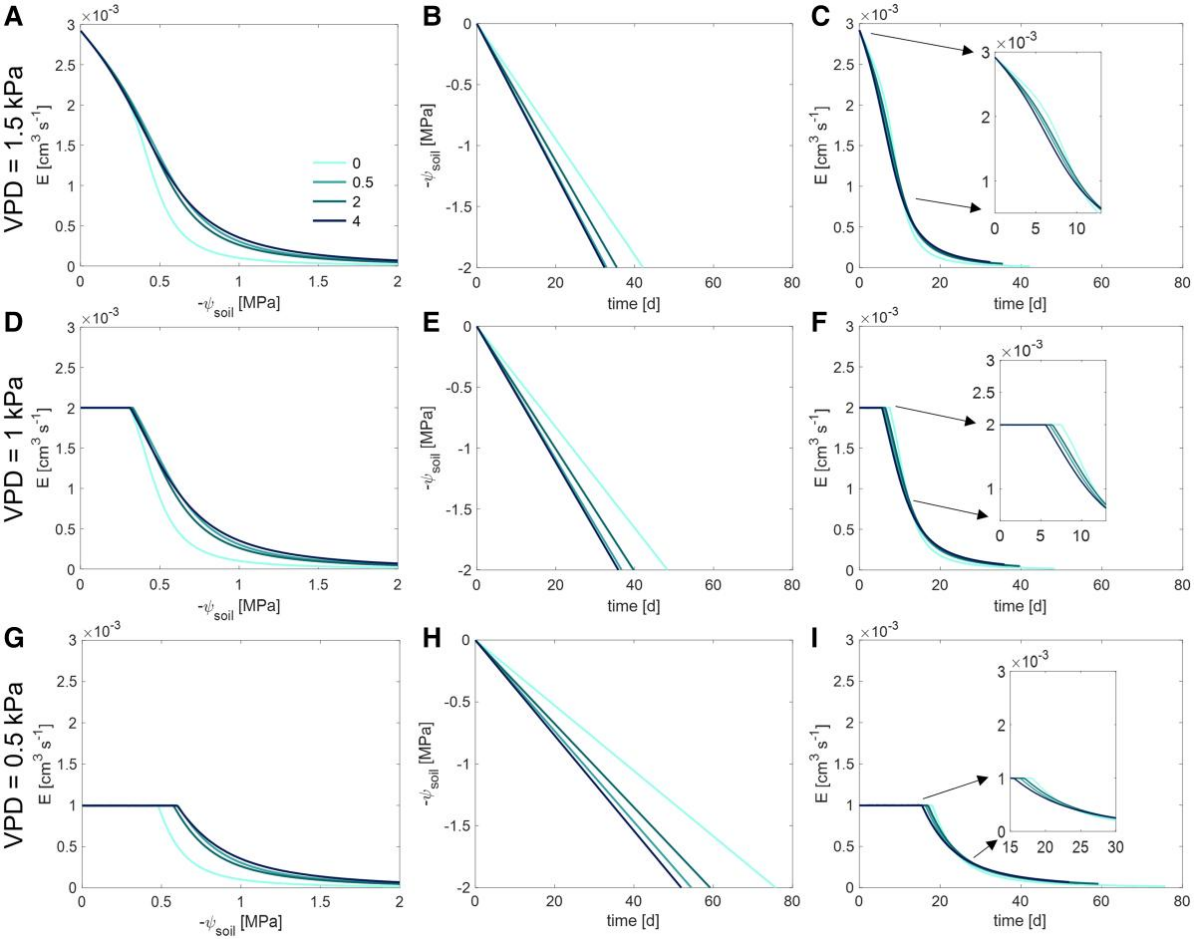
Temporal differences in the decrease in soil matric potential (ψ_soil_) and transpiration rate (*E*) in the loamy soil with 4 mucilage contents (mg mucilage g^−1^ soil) and under 3 VPD conditions (top to bottom, 1.5, 1, and 0.5 kPa). The colors represent different mucilage treatments: 0 (without mucilage), 0.5, 2, and 4 mg (mucilage) g^−1^ (soil). The insets in subplots **C)**, **F)**, and **I)** show magnified views of the initial decline in *E* during the drying period. The statistical significance analysis of the decline in *E* in subplots **C)**, **F)**, and **I)** over drought and at the initial drying period is shown in Fig. 8.

Mucilage enabled plants to maintain transpiration under drier soil conditions (Figs. 3 and  7). At relatively high VPD levels (1.5 kPa), mucilage buffered the reduction in transpiration when the soil matric potential dropped below −0.23 MPa (Fig. 7A). As the soil dried, mucilage maintained the maximum transpiration rates at a given soil matric potential. For instance, at a ψ_soil_ of −0.64 MPa, plants with 4 mg g^−1^ mucilage content had a relatively higher transpiration rate than those with 0.5 mg g^−1^ (Fig. 7A). Similarly, at a ψ_soil_ of −0.45 MPa, the transpiration rate in plants with 4 mg g^−1^ mucilage exceeded that of those with 2 mg g^−1^ (Fig. 7A). As VPD decreased, the soil limit curve for transpiration remained consistent for a given mucilage content, though the maximum transpiration rate decreased (Fig. 7D and G). Although lower VPD levels shifted the onset of soil limitation to lower soil matric potentials, higher mucilage content further delayed this SOL. For instance, at a medium VPD level (1 kPa), transpiration was constrained at −0.31 MPa in the control soil compared to −0.33 MPa in soil with 4 mg g^−1^ mucilage (Fig. 7D). With a relatively lower VPD level (0.5 kPa), this limitation occurred at −0.47 MPa in the control soil, compared to −0.60 MPa in soil with 4 mg g^−1^ mucilage (Fig. 7G).

**Figure 8.**
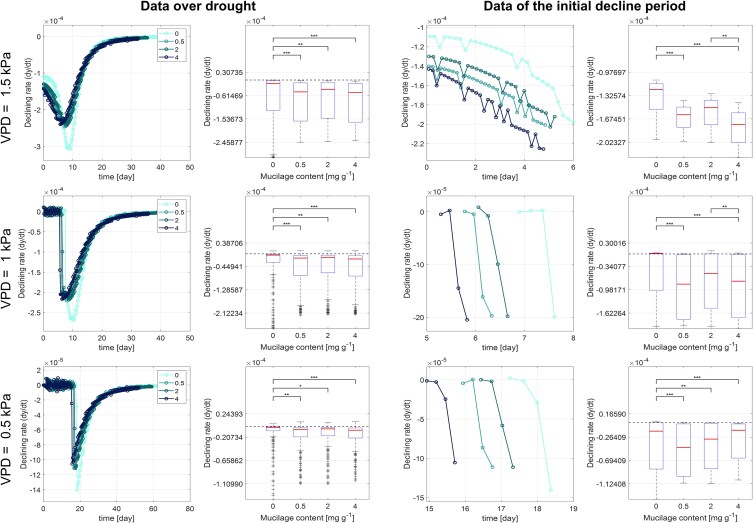
Significant differences in instantaneous declining transpiration rate (d*y*/d*t*) across mucilage treatments and VPD levels during soil drying. The left 2 columns show the instantaneous decline rate over drought (sample size *n* = 200 for all mucilage treatments and VPD conditions), and the right 2 columns present the rate during the initial decline stage (*n* = 31, 4, and 4 for high to low VPD conditions). The rate d*y*/d*t* at each time point was calculated by taking the difference between consecutive measurements and dividing by the time interval. Statistical significance among groups was performed using the Kruskal–Wallis test, followed by the Conover–Iman post hoc test for pairwise comparisons. The asterisk * denote statistical significance (*, *P* < 0.05; **, *P* < 0.01; ***, *P* < 0.001). Center line, median; box limits, upper and lower quartiles; whiskers, 1.5 times interquartile range; +, outlier.

The variation in maximum transpiration rates due to mucilage content influenced the speed at which the soil dried. The increase in mucilage content led to a significant decrease in both soil matric potential (Fig. 7B, E, and H) and transpiration rate (Fig. 7C, F, and I and  Fig. 8), particularly under high VPD levels. Although a lower VPD level attenuated the steepness of the decline in soil matric potential, the accelerating effect of increasing mucilage content on the decrease in soil matric potential persisted (Fig. 7B, E, and H). For instance, under a relatively high VPD level (1.5 kPa), it took 42.1 days for the soil matric potential to decrease from 0 to −2.0 MPa (corresponding to a transition from maximum to minimum transpiration rates) in the control (Fig. 7B and C). In contrast, for plants with 0.5, 2, and 4 mg g^−1^ mucilage, the same decrease in soil matric potential and transpiration occurred in 32.9, 35.5, and 32.4 days, respectively (Fig. 7B and C). Under a low VPD level (0.5 kPa), this duration in the decrease in soil matric potential was prolonged to 75.8, 54.5, 59.2, and 51.9 days with 0, 0.5, 2, and 4 mg g^−1^ mucilage, respectively (Fig. 7H and I).

## Discussion

We investigated the impact of variations in maize root mucilage on soil hydraulic properties and root water uptake during soil drying and increasing VPD. Mucilage enhances soil water retention and attenuates the drop in unsaturated hydraulic conductivity, as well as the gradients in matric potential at the root–soil interface, with more pronounced effects at higher mucilage contents. These modifications maintained higher transpiration rates and expanded the hydroscape zone, thereby affecting plant responses to soil and atmospheric drought.

### Effect of maize root mucilage on soil hydraulic properties

Mucilage significantly reshaped soil hydraulic properties, as evidenced by altered soil water retention and hydraulic conductivity curves (Fig. 2). Previous studies with chia seed mucilage (Xiao et al. 2024; Benard et al. 2019; Ahmed et al. 2014; Naveed et al. 2019) and a recent study with cowpea (Abdalla et al. 2024) showed comparable effects. Aligning with those findings, we showed that maize root mucilage similarly enhanced water retention and mitigated conductivity loss, particularly at lower soil matric potentials, which are crucial for plant water uptake during edaphic stress.

#### Effect of maize mucilage on water retention curve

The capacity of mucilage to enhance water retention depends on both soil texture and mucilage physical properties, especially surface tension and viscosity (see next section). These factors directly affect soil water retention, while the amount of mucilage exuded, and its properties are largely determined by plant species. Collectively, these factors jointly determine the overall effectiveness of mucilage in increasing soil water retention (Kroener et al. 2014, 2018; Adamczewski et al. 2025; Hosseini et al. 2025). In our study, increasing mucilage content from 0.05% to 0.4% retained soil moisture by 2% to 9% at −0.01 MPa and by 1% to 17% at −0.10 MPa (Fig. 2A). This trend of enhanced water retention aligns with chia seed mucilage findings. For instance, Ahmed et al. (2014) observed a large 70% increase in soil moisture at −0.01 MPa and a 100% at −0.10 MPa with 1.25% chia seed mucilage in sandy soils. Moreover, Rosskopf et al. (2022) demonstrated that the addition of 0.2% chia seed mucilage in loamy soil, within the range from −0.006 to −1.50 MPa, retained 19% to 56% more water compared to the control. These results suggest that mucilage might be a plant strategy to moderate the gradient in matric potential at the root–soil interface, and hence maintain water flow during soil and atmospheric drying (Akale et al. 2025). It is worth noting that the effect of mucilage on water retention is highly soil-texture specific, with the effect being more pronounced in coarse-textured soils (Kroener et al. 2018).

#### Effect of maize root mucilage on saturated hydraulic conductivity

Mucilage dramatically changes both saturated (*k*_sat_) and unsaturated (*k*_s_) hydraulic conductivity. Its high viscosity consistently reduces *k*_sat_, with the magnitude of reduction depending on mucilage content and soil texture (Kroener et al. 2018). For example, chia seed mucilage at 0.25% decreased *k*_sat_ by more than 4 orders of magnitude in sandy-loam soil (Benard et al. 2019). Chia seed mucilage is more viscous than maize mucilage (Naveed et al. 2019). Additionally, while our estimation of *k*_sat_ was inferred from unsaturated hydraulic conductivity measurements (which might introduce some uncertainty), the consistent trends across models (Supplementary Fig. S2) indicate that these derived *k*_sat_ values are reasonably robust. In line with this broader pattern, the model predicted that *k*ₛₐₜ decreased by 1 order of magnitude at 0.05% to 0.2% mucilage and by 2 orders at 0.4% (Fig. 2B).

The impact of mucilage on *k*_sat_ is also highly soil-texture specific (Kroener et al. 2018). At 0.4% chia seed mucilage, *k*_sat_ was more than 1,000 times larger in coarse soil compared to silty soil, but this disparity vanished at a concentration of 0.8% as a continuous viscous film dominated pore resistance. At higher concentrations (e.g. 0.8%), mucilage plugged large pores in coarse soils, lowering permeability to levels comparable with finer soils. Thus, at high mucilage concentrations, film continuity rather than soil texture ultimately determines *k*_sat_ (Kroener et al. 2018).

The species-specific properties of mucilage further modulate its effect on *k*_sat_ (Knott et al. 2022; Werner et al. 2022; Berauer et al. 2023). For instance, Naveed et al. (2019) found that the surface tension of maize and barley mucilage decreased with increasing mucilage content, but for chia seeds, it decreased initially before increasing. Overall, the maximum viscosity of maize and barley mucilage at low shear rates (e.g. 0.01 s^−1^) was 2 to 3 orders of magnitude lower than that of chia seed mucilage, with maize mucilage exhibiting approximately twice the viscosity of barley (Naveed et al. 2017 ; Naveed et al. 2019 ). The high viscosity of mucilage impedes water flow primarily because mucilage forms a polymeric network between soil particles, creating a dense, sticky layer that fills soil pores and hinders water movement. The polymer matrix can block large pores or narrow the pore channels, reducing the overall hydraulic conductivity. This reduction, as observed in previous studies, is generally proportional to the viscosity of mucilage (Kroener et al. 2014; Volk et al. 2016). These findings align with our observations that mucilage significantly impacts *k*_sat_, with higher contents leading to greater reductions.

#### Effect of maize root mucilage on unsaturated hydraulic conductivity

The effects of mucilage on unsaturated soil hydraulic conductivity (*k*_s_) are more complex and less straightforward. The impact of mucilage on unsaturated flow can vary depending on mucilage content and/or properties. Our measurements showed that, as soil dried, the drop in *k*_s_ was less pronounced with mucilage addition (Fig. 2B), with *k*_s_ values being higher than the control at around −0.20 MPa. Benard et al. (2019) observed that, in sandy-loam soil amended with chia seed mucilage, the decrease in *k*_s_ below −1 MPa was less marked compared to the control soil. Differences in the decline in *k*_s_ with increasing amounts of mucilage may also be attributed to the contrasting mucilage properties. Specifically, the higher viscosity and the lower surface tension of chia seed mucilage could explain the less pronounced decrease in *k*_s_ compared to the maize mucilage in our study (Zickenrott et al. 2016; Naveed et al. 2017). These inherent properties enhanced the formation of stable polymeric structures that span across the soil pore space and maintain the continuity of the liquid phase in mucilage-amended soils. This sustained film flow enhances unsaturated conductivity, even at low water potentials, when the control soil would normally exhibit significant reductions in *k*_s_. Thus, mucilage improves liquid connectivity across the rhizosphere, which is particularly beneficial in sustaining root water uptake under progressive soil drying, ultimately mitigating the adverse impacts of edaphic stress on plant growth and productivity (Ahmed et al. 2014; Abdalla et al. 2024; Akale et al. 2025).

A similar modification in *k*_s_ has been observed with extracellular polymeric substances (EPS). Biofilm-forming bacteria were shown to buffer the decline in *k*_s_ during soil drying (Volk et al. 2016), a process likely driven by EPS-induced stabilization of soil aggregates and hydration films. For instance, Zheng et al. (2018) found that EPS from plant growth-promoting rhizobacteria enhanced *k*_s_ more effectively in fine-textured soils (clay soil) than in sandy soil. This effect is attributed to improved pore connectivity and water film continuity, along with altered physicochemical properties, such as surface tension and viscosity (Benard et al. 2019; Nazari et al. 2022). Similarly, the high water-holding capacity of mucilage increases *k*_s_ at negative soil matric potential, possibly facilitating root water uptake as soils dry (Ahmed et al. 2014; Akale et al. 2025; see the next sections). This finding underscores the potential of mucilage as an effective trait for enhancing water retention and improving soil hydraulic properties in the root zone, especially under drought conditions.

### Effect of maize mucilage on matric potential at the root–soil interface

Under high transpiration rates (high VPD), mucilage significantly reduced the diverge of water potential at the soil–root interface (ψ_soil_root_) from the bulk soil matric potential (ψ_soil_) (Fig. 5), highlighting mucilage’s capacity to buffer the drop in matric potential at the root–soil interface (Carminati et al. 2011; Abdalla et al. 2024; Ahmed et al. 2014, 2018a). Similar to the findings of Zarebanadkouki et al. (2022), who observed that amorphous silica attenuated the matric potential drop, our study confirms that substances enhancing water retention and hydraulic conductivity can markedly soften the gradients in matric potential across the rhizosphere. While reduced VPD lowers overall transpiration demand and alleviates water stress, it does not fundamentally change the hydraulic gradient at the soil–root interface when other factors (e.g. soil texture, root length density, and stomatal conductance) are controlled. This suggests the consistency of mucilage as an adaptive trait, especially under water-limited environments (Ahmed et al. 2018a).

By decelerating the sharp drop in matric potential near roots, mucilage delays the onset of hydraulic limitations to transpiration, effectively sustaining water uptake even as the soil dries (Abdalla et al. 2024; Akale et al. 2025). This buffering broadens the hydroscape, enabling operation over a wider range of soil water potentials and promoting anisohydric tendencies (Fig. 6). The mucilage buffering effects across atmospheric conditions, as well as its ability to enhance rhizosphere hydraulic conductivity, particularly at more negative matric potentials, suggest that plants with high mucilage production may exhibit greater resilience to edaphic and atmospheric stresses (Akale et al. 2025). However, these benefits likely vary with soil texture and mucilage physicochemical properties. It should be noted that producing high quantities of mucilage may come at a cost of reduced root/plant growth (Santangeli et al. 2024), and quantifying this potential trade-off remains an important objective for future research. Declining soil–root hydraulic conductance has been identified as a key driver of stomatal closure, as it constrains water flux and prompts plants to reduce transpiration to preserve their water status (Carminati and Javaux 2020; Carminati et al. 2020; Abdalla et al. 2021, 2022; Cai et al. 2022; Rodriguez-Dominguez and Brodribb 2020). Therefore, mucilage, in enhancing rhizosphere hydraulic conductivity, is crucial for effective plant water-use regulation and overall drought resilience (Ahmed et al. 2018a).

### Effect of maize mucilage on isohydricity and stomatal regulation under different VPD levels

The modification of mucilage to soil hydraulic properties profoundly influences the soil–plant hydraulic continuum, driving key changes in stomatal regulation and water-use strategies. By amplifying the nonlinearity of unsaturated hydraulic conductivity (Fig. 2B), mucilage shifted the SOL and reshaped the hydroscape area, which is suggested as a critical determinant of isohydricity (Fig. 6). By modifying below-ground hydraulic dynamics, mucilage mediates a shift in plant behavior, particularly under edaphic stress (Carminati et al. 2011; Ahmed et al. 2014; Schwartz et al. 2016; Abdalla et al. 2024). These consequences are critical for plant adaptation, as they prolong the sustained transpiration period, allowing plants to utilize available water more efficiently (Carminati et al. 2011 ; Abdalla et al. 2024; Akale et al. 2025).

In our simulation, mucilage effectively delayed the transition from a linear to a nonlinear hydraulic response (Fig. 3), allowing higher transpiration rates and lower midday leaf water potentials (ψ_leaf_MD_). This broadened the hydroscape zone and reduced stomatal sensitivity to declining soil matric potential (Fig. 6). By mitigating the decline in unsaturated hydraulic conductivity, mucilage buffers the gradients in matric potential at the root–soil interface and facilitates root water uptake, especially under relatively dry soil conditions. These effects align with previous findings by Knipfer et al. (2020) and Javaux and Carminati (2021), which highlighted the critical role of soil and root hydraulic conductance in influencing stomatal responses during drought (Manandhar et al. 2024), and demonstrate how mucilage prolongs transpiration and optimizes plant water-use strategies under edaphic and atmospheric stress (Akale et al. 2025).

The ψ_leaf_MD_ − ψ_soil_ relationship reflects the interplay between stomatal regulation and hydraulic resistance, with its slope serving as a proxy for isohydricity (Meinzer et al. 2016; Charrier 2020; Knipfer et al. 2020; Javaux and Carminati 2021). The ψ_leaf_MD_ decline in mucilage-enriched soils indicates a shift toward anisohydric behavior (Fig. 6), where plants tolerate relatively lower leaf water potentials during soil drying. This shift enhances root water uptake and extends the period of transpiration during progressive soil drying. The broader hydroscape is consistent with reduced soil–plant hydraulic resistance (Figs. 3 and 4), as predicted by Javaux and Carminati (2021). Additionally, mucilage enhances anisohydric tendencies by extending the phase of soil water availability and reducing the sensitivity of stomata to hydraulic signals. By permitting stomata to remain open at lower leaf water potentials, anisohydric behavior might increase the risk of xylem embolism and hydraulic failure (Benson et al. 2022). This trade-off may be advantageous during short-term or terminal drought. However, its efficacy diminishes as soil keeps drying, eventually surpassing the hydraulic benefits of mucilage under prolonged soil and atmospheric drying (Fig. 6).

Charrier et al. (2018) linked stomatal behavior to environmental variables like VPD. Intuitively, as plant water demand increases, hydraulic conductance in the rhizosphere decreases due to localized soil water depletion around roots. Moreover, under high VPD, root shrinkage can reduce root–soil contact and create gaps, hindering hydraulic continuity (Huck et al. 1970; Faiz and Weatherley 1982; Duddek et al. 2022, 2023), thus potentially triggering stomatal closure. Our simulations showed that mucilage modulated the root-zone environment, mitigating these effects and modulating stomatal responses to increasing VPD. This buffering effect promoted more anisohydric behavior, especially under high mucilage contents, where plants could tolerate relatively lower ψ_leaf_MD_ without compromising hydraulic safety (Fig. 6). Although maximum transpiration rates were reduced under relatively lower VPDs (0.5 and 1 kPa), the positive impact of mucilage on root water uptake was preserved (Fig. 3). It was shown that abscisic acid (ABA) synthesis in leaves under both soil drying and high VPD can also initiate stomatal closure ahead of purely hydraulic signals. While our current model does not explicitly simulate ABA signaling nor an explicit leaf-level VPD response, and hence stomatal dynamics arise solely from hydraulic feedbacks. Future model enhancements could couple a simple ABA-production and guard-cell sensitivity module to further incorporate these hormonal dynamics. Under lower VPD levels, sustained transpiration in drier soils suggests the potential of mucilage to enhance adaptability to fluctuating climates, aligning with Nazari et al. (2020), who found a highly positive correlation between mucilage exudation and the native VPD of the genotype agroecological zones. This pattern may also explain why higher mucilage amounts enable plants to maintain much lower leaf water potential under drier soil conditions (Fig. 6).

### Effect of maize mucilage on plant water-use strategy

The adaptive strategy induced by mucilage revealed a trade-off. While higher mucilage contents postpone the onset of water limitation, they also accelerate the rate at which soil matric potential declines and shorten the duration of water availability (Fig. 7). This accelerated drying implies that while mucilage enhances immediate water uptake and buffers hydraulic limitations, it may also lead to a faster depletion of soil moisture reserves during prolonged drought. Consequently, plants may face an earlier onset of severe water stress once soil moisture is exhausted, potentially limiting long-term drought resilience. In competitive fields, this can be advantageous: mucilage-rich plants extract moisture from drying soil more effectively than neighbors with low or no mucilage, thereby depleting the local water supply ahead of them and maintaining better water status during the drying period, and therefore increasing their chances of surviving that drought episode.

The implication of this mucilage-induced trade-off is not straightforward. On the one hand, mucilage serves as an effective hydraulic buffer in the initial stages of soil drying. By reducing *k*_sat_ (Fig. 2), it suppresses vertical drainage and retains more water in the immediate root zone. For instance, Akale et al. (2025) compared 2 cowpea genotypes (with contrasting mucilage production rates) on plant response to soil and atmospheric drought. The authors found that, under wet conditions, the high mucilage genotype conserves water by restricting transpiration at a lower VPD, and during soil drying, it maintains transpiration longer than the low mucilage genotype by preserving hydraulic continuity between the roots and soil. Within this moist rhizosphere, mucilage potentially maintains close soil–root contact and a moist micro-environment. The result is a faster local drawdown of soil water potential, as roots rapidly deplete the mucilage-retained water in the rhizosphere. Nevertheless, this moist rhizosphere “micro-reservoir” still enables plants to sustain transpiration and photosynthesis during intermittent dry spells. This capability is particularly advantageous in environments characterized by fluctuating water availability, such as those with intermittent rainfall, where plants capitalize on periods of moisture replenishment. The functional benefit of mucilage emerges as context-dependent, with its efficacy directly influenced by environmental conditions and soil textures (Kroener et al. 2018; Berauer et al. 2023). On the other hand, the accelerated decline in soil matric potential associated with higher mucilage contents highlights a potential vulnerability under extended drought. Therefore, while mucilage-mediated hydraulic buffering enhances short-term drought resistance, it may require careful management to balance immediate water uptake benefits against the risk of rapid soil moisture depletion (Tardieu 2012; Ahmed et al. 2018a; Tardieu et al. 2018).

### Limitations

While this study offers important insights into the role of mucilage in plant hydraulics and soil–plant water relations, we are also aware of certain limitations. In the simulations, we assumed constant mucilage concentrations during soil drying, thereby omitting dynamic processes like decomposition (Ahmed et al. 2018b; Niedeggen et al. 2024). Although this simplification helped isolate the effects of mucilage, it may not fully reflect field or long-term conditions, where these dynamics could influence outcomes. Notably, Abdalla et al. (2024) found that cowpea mucilage delayed hydraulic limitations to transpiration during soil drying, where mucilage dynamics were covered in the growing period (approximately 1 month). Moreover, our focus on maize root mucilage involved measurements and simulations conducted exclusively in a loamy soil as a compromise due to experimental constraints. Although this approach allowed for a controlled and detailed investigation of the mucilage influence, it does not capture the variability of mucilage effects across different soil types, as demonstrated by Kroener et al. (2018). Future research that includes multiple soil textures would enhance our understanding of mucilage impacts on soil–plant water relations across diverse environments.

Additionally, we emphasize that these conclusions were drawn exclusively from simulations assuming uniform environmental conditions, and thus involve inherent limitations. In real-field environments, significant spatial and temporal variability in soil moisture, temperature, and VPD can influence plant hydraulic responses in ways not captured by our model. To validate these findings and assess their broader applicability, field experiments under natural conditions are essential. Such studies would help elucidate the implications of mucilage-mediated hydraulic responses across varying agroecological conditions.

## Concluding remarks

This study demonstrated mucilage's crucial role in plant water relations by modifying soil hydraulic properties, root-zone water potential dynamics, stomatal regulation, and plant water-use strategies under varying VPD conditions. Mucilage significantly enhanced soil water retention, thereby reducing the saturated hydraulic conductivity, yet attenuating the drop in unsaturated hydraulic conductivity during soil drying. These shifts delayed the onset of hydraulic limitations as soil dried and enhanced soil–plant hydraulic conductance, particularly at higher mucilage contents, independent of the VPD level. This enables plants to maintain higher transpiration rates at more negative leaf water potentials, especially in drying soils. Consequently, plants exhibited more anisohydric behavior. This adaptive response supports continued water uptake and stomatal function during early soil drying. A trade-off being that while mucilage delayed water limitation through enhanced soil–plant hydraulic conductance and anisohydry, higher mucilage contents accelerated the rate of soil drying, leading to a faster decline in soil matric potential and transpiration rates during drought. Thus, the presence of mucilage may effectively shorten the duration over which plants sustain water uptake during prolonged drought.

In summary, mucilage plays a dual role in plant water-use strategies under drought. It enhances immediate drought resistance by stabilizing water uptake and mitigating hydraulic stress, yet it may lead to faster soil moisture depletion under extended drought conditions. These findings emphasize the complexity of mucilage in soil–plant hydraulic interactions and its implications for plant adaptation to drought stress. Future studies should therefore experimentally explore mucilage effects across diverse soil textures and plant species, and assess the long-term trade-offs between improved water uptake and accelerated soil drying, to fully harness the potential of mucilage in improving drought resilience in agricultural and natural systems.

## Materials and methods

### Mucilage and soil preparation

Several hybrid maize (*Z. mays*) genotypes obtained from KWS Saat SE & Co. KGaA were used for this study (see Supplementary Table S1). The genotypes of plants were grown in a corn field, and mucilage samples were obtained by visually selecting aboveground nodal roots at the culmination of tassel emergence (BBCH 59), following the protocol described by Ahmed et al. (2015). Briefly, the mucilage-laden roots were excised from the stem and transported to the laboratory, where they were gently cleaned of soil and plant residues using distilled water and a coarse sieve. Thereafter, the root samples were rinsed overnight with distilled water to facilitate mucilage hydration. The following day, the excess water was removed by decantation over a sieve with a pore size of 200 *µ*m (ATECHNIK GmbH, Germany), and the hydrated mucilage was collected in a 20 ml beacon using a syringe. The collected mucilage was immediately frozen at −21 °C to prevent degradation. The frozen-stored mucilage was thawed, filtered through a 100 *μ*m stainless steel sieve to remove impurities, and then freeze-dried to enhance soil integration.

Loamy soil, classified as a haplic Phaeozem under agricultural use, was prepared following the methods described by Vetterlein et al. (2021). Initially, the soil was sieved using a heavy-duty vibrating screen with mesh sizes of 40 and 20 mm. Subsequently, the soil was further sieved through a 2 mm mesh. Visible roots and other organic materials were removed manually. The prepared soils were thoroughly mixed, and 60 g of soil was weighed for each treatment. The mucilage content to be incorporated was determined based on previous studies and the available amount of mucilage. For instance, Zickenrott et al. (2016) estimated that realistic mucilage contents in the rhizosphere range between 0.05 and 5 mg of dry mucilage per gram of dry soil. Accordingly, 3 mucilage concentrations were selected: 0.5, 2, and 4 mg of dry mucilage per gram of dry soil, with 3 replicates per treatment. The desired mucilage content was achieved by accurately weighing the required amount and gently mixing it with the soil using a mortar and pestle, without applying excessive pressure. A control treatment without mucilage was prepared under identical conditions.

### Measurement of soil hydraulic properties with mucilage

The soil hydraulic properties were assessed using the evaporative method implemented in the HYPROP system (Meter Group, Munich, Germany). To minimize the volume of soil and ensure efficient use of the limited mucilage sample, custom-made columns with a volume of 29.3 ml were used. These columns were inserted into a 250 ml sample ring and packed to a target bulk density of 1.39 g cm^−3^. The packed samples within the ring were saturated and then allowed to dry via evaporation from the top, while the mass was monitored with a precision HYPROP scale unit (2,000-g range; 0.01-g resolution). Additionally, 2 precision mini-tensiometers were inserted at depths of 4.3 cm (for the larger tensiometer) and 1.7 cm (for the smaller tensiometer) to measure water potential. The time between the start of the measurement and the point of air entry into the bottom tensiometer was defined as the drying duration and was recorded using the software LABROS SoilView Analysis (METER Group). After the measurement, the samples were oven-dried at 105 °C for 24 h to determine the dry weight and, consequently, the volumetric water content.

To assess the soil hydraulic properties at lower water potentials, 5-g soil subsamples were collected immediately after mixing the soil and mucilage to prevent degradation during HYPROP measurement. These subsamples were weighed in stainless steel cups designed for measurement with the WP4C Dewpoint Potential Meter (METER Group, Munich, Germany). The bottom of the cup was completely covered, while the sample cup was filled to no more than half its capacity. The samples were hydrated with approximately 2 ml of tap water and allowed to equilibrate over time. If the water content was too high and the measurement could not be conducted, the open samples were set aside and re-measured at a later time. When all measurements were completed, the samples were oven-dried at 105 °C to determine the dry weight. Measurement data from both the WP4C and the HYPROP systems were combined for each soil type and mucilage content. Subsequently, the Brooks and Corey model was applied to simulate soil water retention and hydraulic conductivity curves based on the HYPROP data.

### The soil–plant hydraulic model

We employed a simplified soil–plant hydraulic model, developed by Carminati and Javaux (2020), to simulate the water flow through the soil–plant continuum, with a focus on capturing the dynamic water potential gradients in the soil under different mucilage contents. This model specifically addresses how water potential gradients evolve under varying transpiration rates and soil moisture conditions. It operates under the assumption of steady-state water flow and incorporates a series of resistances across different components: the soil, the soil–root interface, the root xylem, and the aboveground xylem. The model simplifies the plant structure by representing it as a single active root for water uptake, allowing for the simulation of water movement from the soil, through the root system and xylem, and up to the leaf.

#### Radial water flow in soil

The radial water flow in the soil toward the root surface is described, assuming a radial geometry around the root tip. The radial water flux toward the root is described by:


(1) $$q=-k_{s}\;(\psi_{m})\frac{\partial \psi_{m}}{\partial r}$$

where *q* is the water flux (cm s^−1^), *k*_s_ is the soil hydraulic conductivity (cm s^−1^), ψ_m_ is the soil matric potential (hPa, used in the simulation and converted to MPa in the results), and *r* is the radial distance (cm). *k*_s_ is parameterized using the Brooks and Corey model:


(2) $$k_{s}(\psi )=k_{\mathrm{sat}}{\left(\frac{\psi_{m}}{\psi_{0}}\right)}^{\tau }$$

where *k*_sat_ is the saturated hydraulic conductivity (cm s^−1^), ψ_0_ is the air entry value (cm), and *τ* is a fitting parameter (−).

The boundary conditions include no flow at the outer radius of the soil (*r*_b_) and a uniform flux at the soil–root interface:


(3) $$q(r_{0})=E/2\pi r_{0}L_{\mathrm{act}}$$

where *r*_0_ is the root radius (cm), *E* is the transpiration rate (cm^3^ s^−1^), and *L*_act_ is the root length active in water uptake (cm). The outer radius *r*_b_ is calculated from the soil volume *V* (cm^3^) and *L*_act_: *r*_0_ = (*V*/π*L*_act_)^0.5^.

#### Water flow through the root and xylem

The water flow in the root is given by:


(4) $$E=K_{root}(\psi_{\mathrm{soil}\_\mathrm{root}}\text{–}\psi_{\mathrm{xylem}\_\mathrm{root}})$$

where *K*_root_ is the root hydraulic conductance (cm^3^ s^−1^ hPa^−1^), ψ_soil_root_ is the water potential at the soil–root interface (hPa), and ψ_xylem_root_ is the water potential at the xylem collar (hPa).

In the xylem, the flow is defined as:


(5) $$E=K_{x}(\psi_{\mathrm{xylem}\_\mathrm{root}}\text{–}\psi_{\mathrm{leaf}})$$

where *K*_x_ is the xylem conductance of the shoot (cm^3^ s^−1^ hPa^−1^), and ψ_leaf_ is the leaf xylem pressure (hPa). The xylem conductance *K*_x_ is adjusted for cavitation using:


(6) $$K_{x}=K_{\mathrm{root}}{\left(\frac{\psi_{\mathrm{leaf}}}{\psi_{0x}}\right)}^{-\tau_{x}}$$

where ψ_0x_ is the xylem pressure at which *K*_x_ starts to decrease (hPa), and τ_x_ is a fitting parameter (−). The plant hydraulic conductance *K*_plant_ (cm^3^ s^−1^ hPa^−1^), which is the harmonic mean of *K*_root_ and *K*_x_, approximates *K*_root_ when *K*_x_ >> *K*_root_, assuming no cavitation.

The total soil–plant hydraulic conductance *K*_sp_ is given by:


(7) $$K_{\mathrm{sp}}=E/(\psi_{\mathrm{Soil}}\text{–}\psi_{\mathrm{leaf}})$$

and it can be related to the individual conductances:


(8) $$\begin{array}{r} 1/K_{\mathrm{sp}}=1/K_{\mathrm{soil}}+1/K_{\mathrm{plant}}=1/K_{\mathrm{soil}}+1/K_{\mathrm{root}}+1/K_{x}\;\;1/K_{\mathrm{soil}}+1/K_{\mathrm{root}} \end{array}$$

where *K*_soil_ is calculated as: *K*_soil_ = *E*/(ψ_soil_ − ψ_soil_root_). The model allows for the prediction of changes in leaf water potential as soil moisture varies and transpiration rates increase, providing insights into the limits of hydraulic function under different conditions of atmospheric demand.

#### SOL, simulation, and analysis

This model was used to investigate how water potential in the soil–plant continuum responds to varying transpiration rates. It focuses on identifying the onset of hydraulic limitation, defined as the point where the slope of the transpiration rate (*E*) versus leaf water potential (ψ_leaf_) curve declines to 60% to 80% of its maximum value. This SOL represents the maximum transpiration rate that can be sustained before the risk of hydraulic failure increases significantly, SOL(ψ_soil_) = $\frac{\partial E}{\partial \psi_{\mathrm{leaf}}}|$ ψ_soil_. The model shows that the slope of the *E*(ψ_leaf_) relationship is maximal at zero transpiration and decreases as transpiration increases, reflecting a transition from linear to nonlinear water flow behavior with soil drying. By examining the interplay between soil drying and plant water uptake, the model offers a detailed framework for understanding how soil and root hydraulic properties affect transpiration and leaf water potential.

For each mucilage treatment, we parameterized the Brooks and Corey hydraulic functions by fitting the model to experimentally determined water retention data (θ(Ψ)) and *K*ₛₐₜ measurements. These parameter sets replace the standard soil parameters in the Richards' equation solver (Equations 1 and 2), thereby capturing the direct impact of mucilage on both retention and unsaturated conductivity within the model framework. We acknowledge that mucilage exudation in situ is a dynamic, root-, age-, and environment-dependent process. Here, we employ a static treatment to represent total mucilage accumulation.

In the simulations, 3 VPD levels—1.5 kPa (high), 1 kPa (medium), and 0.5 kPa (low)—were considered to explore the transpiration response of the plants exuding different mucilage amounts under contrasting atmospheric water demand conditions. These thresholds align with the 0.5 to 1.5 kPa mean daytime VPD range globally (e.g. ERA5 data, https://cds.climate.copernicus.eu/), where 1 kPa reflects typical field conditions, and 1.5/0.5 kPa represent elevated/reduced evaporative demand. By assuming equal growth status (in terms of leaf area and root length) across different atmospheric conditions, we simplified the model to focus solely on the role of mucilage exudation in regulating plant water uptake and stomatal behavior. This approach allows us to isolate the impact of mucilage content on plant hydraulic responses, minimizing confounding factors such as plant size or root system variability. By controlling for these variables, we can more effectively assess how mucilage influences soil water availability, hydraulic properties, water uptake, and stomatal regulation. This simplification improves our capacity to explore the adaptive role of mucilage in maintaining water uptake and regulating stomatal conductance in response to changing atmospheric conditions imposed by varying VPD levels.

### Statistical analysis

We used nonparametric tests throughout because the data violated normality and homoscedasticity. First, we assessed overall treatment and method effects with Kruskal–Wallis tests (α = 0.05), then applied Conover–Iman rank-based pairwise comparisons with Holm adjustment to identify which treatment and method pairs differed significantly (*P* < 0.05). Pairwise treatment differences in slope were tested using paired comparisons across points. Differences were considered statistically significant when the 95% confidence intervals (CIs) of the mean pairwise differences excluded zero. Statistical comparisons were performed in MATLAB (MathWorks Inc., R2022b).

## Supplementary Material

kiaf510_Supplementary_Data

## Data Availability

The data underlying this article are available within the article.

## Supplementary data

The following materials are available in the online version of this article.

**Supplementary Figure S1.** Significant differences in soil hydraulic properties between mucilage treatments from measurements and fittings.

**Supplementary Figure S2.** Saturated soil hydraulic conductivity (*k*_sat_) of the soil with different mucilage contents fitted by different hydraulic conductivity models.

**Supplementary Figure S3.** Comparison of the relationship between soil matric potential (ψ_soil_) and matric potential at the soil–root interface (ψ_soil_root_) at transpiration rate (*E*) of 0.001 cm^3^ s^−1^ under different VPD conditions.

**Supplementary Table S1.** Hybrid maize (*Z. mays*) genotypes used in this study.

**Supplementary Text.** Description of hydraulic conductivity models used in Supplementary Fig. S2.

## Dive Curated Terms

The following phenotypic, genotypic, and functional terms are of significance to the work described in this paper:

water CHEBI: CHEBI:15377
