# Supplementary material for: Maize root mucilage alters stomatal responses to soil and atmospheric drought: Implications for plant water use
Source: Plant Physiol. 2025 Oct 13;199(3):kiaf510. doi: 10.1093/plphys/kiaf510 (PMC12585534; doi:10.1093/plphys/kiaf510)
Supplement: kiaf510_Supplementary_Data [file kiaf510_supplementary_data.pdf]

Supplemental Data

1. Supplemental Table

Supplementary Table S1. Hybrid maize (*Zea mays*) genotypes used in this study. The seeds were obtained from KWS Saat SE & Co. KGaA.

Genotypes	Domestication type
30V92	Hybrid
900MGOLD	Hybrid
DHO2	Hybrid
DHO4	Hybrid
KALUMET	Hybrid
KAPITOLIS	Hybrid
KASHIMIR	Hybrid
KENTOS	Hybrid
KERAVNOS	Hybrid
KIDEMOS	Hybrid
KLEOPATRAS	Hybrid
KWS 2376	Hybrid
KWS KERUBINO	Hybrid
KWS SMARAGD	Hybrid
KXB7471	Hybrid
KXB7554	Hybrid
KXB8383	Hybrid
KXB8386	Hybrid
P3522	Hybrid

2. Supplemental Figures

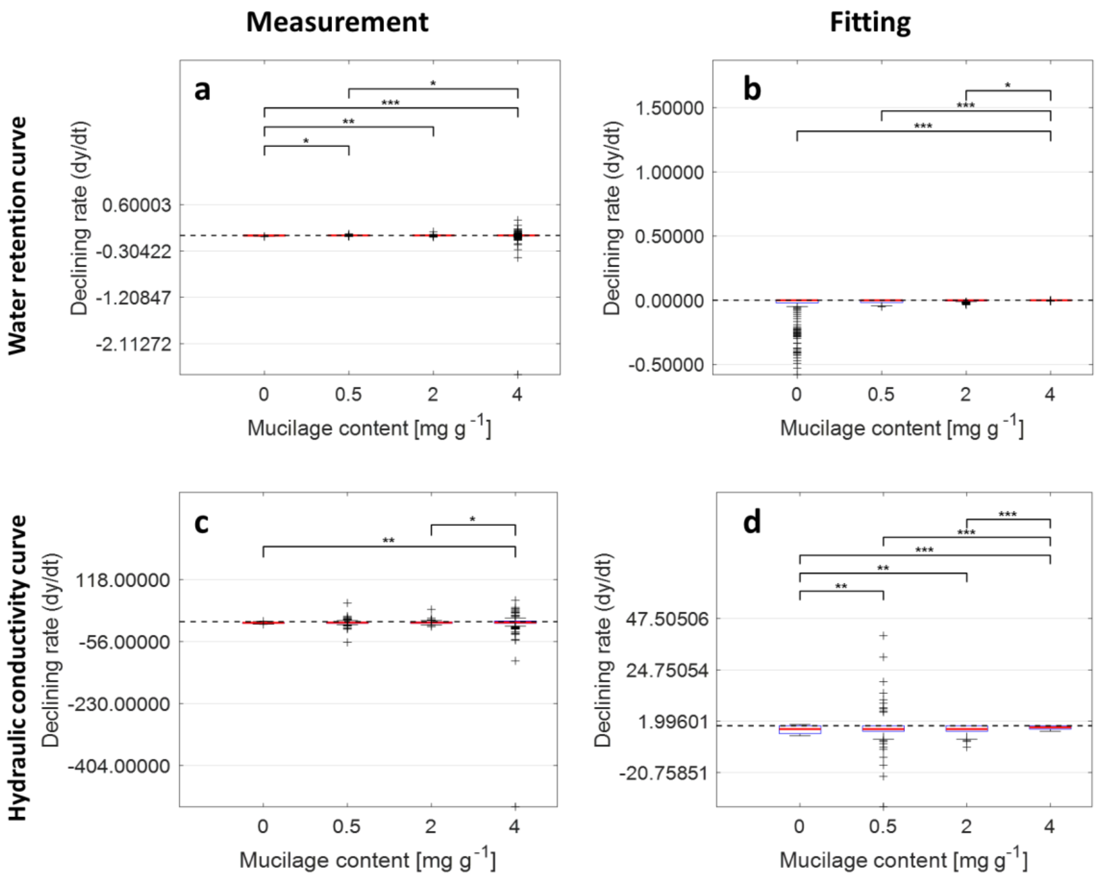

Supplementary Figure S1 Significant differences in soil hydraulic properties between mucilage treatments from measurements and fittings. (**a - b**) Comparison of the decline rate (dy/dt) in soil moisture at each soil matric potential from measurement and fittings, respectively. (**c - d**) Comparison of the decline rate (dy/dt) in soil hydraulic conductivity at each soil matric potential from measurement and fittings, respectively. Decline rate (dy/dt) was calculated by taking the difference between consecutive soil moisture (or hydraulic conductivity) measurements (or fittings) and dividing by the soil matric potential interval. Soil matric potential and hydraulic conductivity were processed in logarithm format. The sample sizes (n) were n = 69, 128, 148, and 273 (due to the distribution of measurement points) for mucilage contents of 0, 0.5, 2, and 4 mg g^{-1} in subplot **a** and **c**, respectively, while a constant sample size n = 201 was used for all treatments in subplot **b** and **d**. Statistical significance among groups was performed using the Kruskal-Wallis test, followed by Conover-Iman post-hoc test for pairwise comparisons. The asterisk * denote statistical significance (*, $p < 0.05$; **, $p < 0.01$; ***, $p < 0.001$). Center line, median; box limits, upper and lower quartile; whiskers, 1.5 times interquartile range; +, outlier.

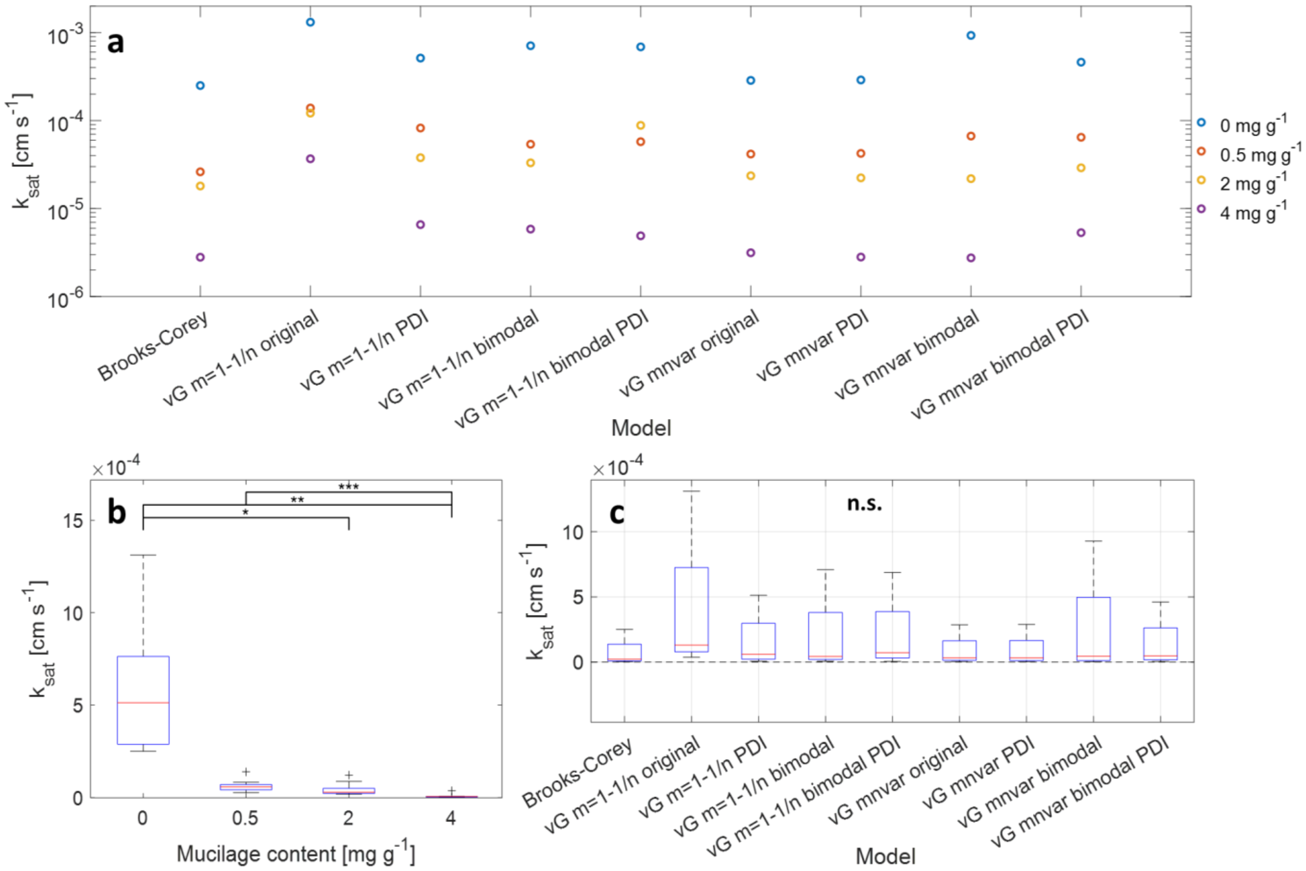

Supplementary Figure S2 Saturated soil hydraulic conductivity (k_{sat}) of the soil with different mucilage contents fitted by different hydraulic conductivity models. (**a**) Comparison of k_{sat} fitted by nine models. (**b**) Comparison of k_{sat} differences across four mucilage treatments with significance annotations. (**c**) Comparison of k_{sat} differences across nine models with significance annotations. The sample sizes in subplot **b** and **c** are 9 and 4, respectively. Statistical significance among groups was performed using the Kruskal-Wallis test, followed by Conover-Iman post-hoc test for pairwise comparisons. The asterisk * denote statistical significance. n.s.: not significant. Center line, median; box limits, upper and lower quartile; whiskers, 1.5 times interquartile range; +, outlier. **vG**: van Genuchten. $\boldsymbol{m = 1 - 1/n}$: the variable m is constrained. **mnvar**: the variable m is unconstrained. **original**: the hydraulic conductivity function proposed by van Genuchten (1980). **PDI**: the unimodal PDI van Genuchten function modified by Peters (2013, 2014), Iden and Durner (2014). **bimodal**: the bimodal van Genuchten function modified by Durner (1994). **bimodal PDI**: the bimodal van Genuchten function modified by Iden and Durner (2014) and Peters (2014). The detailed hydraulic conductivity models are described below (see **Supplemental Text**).

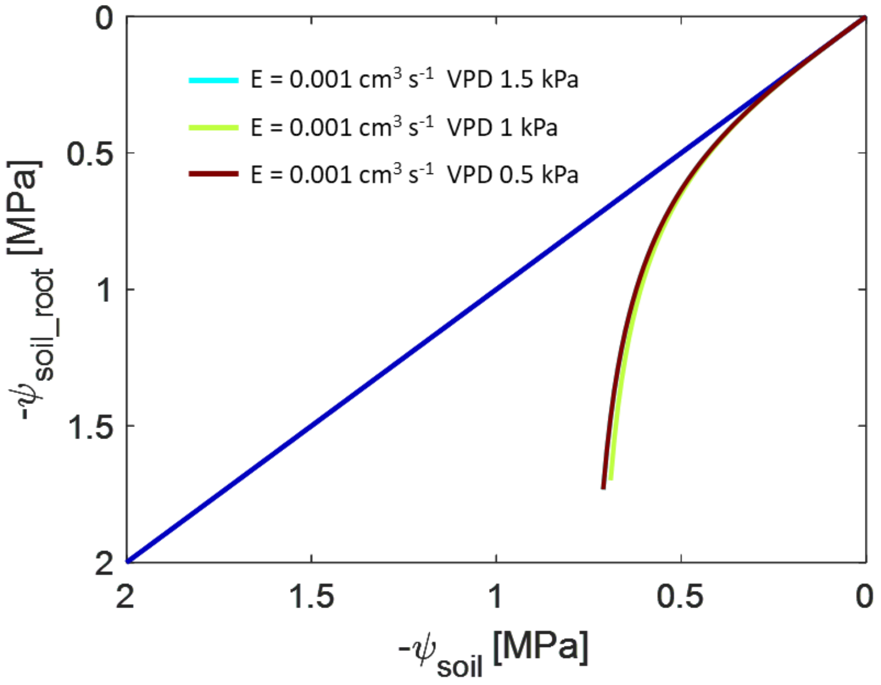

Supplementary Figure S3 Comparison of the relationship between soil matric potential (ψ_{soil}) and matric potential at the soil-root interface (ψ_{soil_root}) at transpiration rate (E) of 0.001 cm^3 s^{-1} under different vapor pressure deficit (VPD) conditions. The three $\psi_{soil} - \psi_{soil\_root}$ curves are from **Fig. 6a, 6e, and 6f**.

3. Supplemental Text

Description of hydraulic conductivity models used in Supplementary Figure S2

1. Original van Genuchten (1980)

The classic model for hydraulic conductivity with coupled parameters m and n.

Hydraulic Conductivity:

$$K(S_e) = K_{sat} \cdot {S_e}^{l} \left[1 - \left(1 - S_e^{1/m}\right)^{m}\right]^{2}$$

where:

- K: unsaturated soil hydraulic conductivity,

- K_{sat}: saturated hydraulic conductivity,

- $S_e = \frac{\theta - \theta_r}{\theta_s - \theta_r}$, S_e: effective saturation,

- θ: Volumetric water content at suction h,

- θ_s: Saturated water content,

- θ_r: Residual water content,

- l: exponent,

- m, n: shape parameter,

with m constrained: $m = 1 – 1/n$; without m constrained, m is independent of n.

Reference: van Genuchten (1980).

2. Unimodal PDI Model

Total conductivity combines capillary, film, and vapor components.

Hydraulic Conductivity:

$$K(h) = K_s\left[\omega K_{\text{cap}}(h) + (1 - \omega)K_{\text{film}}(h) + K_{\text{vap}}(h)\right]$$

where:

- ω is the weighting factor for capillary conductivity,

- $K_{\text{cap}}(h)$ is the capillary conductivity,

- $K_{\text{film}}(h)$ is the film conductivity,

- $K_{\text{vap}}(h)$ is the vapor conductivity.

The capillary conductivity $\boldsymbol{K}_{\textbf{cap}}(\boldsymbol{h})$ is described by the Mualem model:

$$K_{\mathrm{cap}}(h) = K_s\left[S_{\mathrm{cap}}(h)\right]^s \left[\int_{h_0}^{h} \frac{1}{h'}\, dS_{\mathrm{cap}}(h')\right]^2$$

where:

- s: tortuosity and connectivity parameter,

- relative Capillary Saturation S_{cap} is defined: $S_{\mathrm{cap}}(h) = \frac{\Gamma(h)-\Gamma(h_0)}{1-\Gamma(h_0)}$,

- capillary saturation function $\Gamma(h)$: $\Gamma(h) = \left[\frac{1}{1+(\alpha|h|)^n}\right]^m$, $m = 1 - \frac{1}{n}$,

- h_0: suction at oven dryness.

The film conductivity $\boldsymbol{K}_{\mathbf{film}}(\boldsymbol{h})$ is given by:

$$K_{\mathrm{film}}(h) = K_s\left(\frac{h}{h_a}\right)^{-1.5}\left[1 - S_{\mathrm{ad}}(h)\right]$$

where:

- relative adsorbed saturation $S_{\mathrm{ad}}(h)$: $S_{\mathrm{ad}}(h) = 1 - \frac{1}{1+\exp\left(\frac{\log_{10}(h)-\log_{10}(h_a)}{b}\right)}$,

- h_a is the suction below which $S_{\mathrm{ad}}(h) = 1$,

- b is a smoothing parameter and it can be expressed as: $b = 0.1 + 0.2 \cdot \frac{n-1}{n+1} \cdot \exp\left(-\frac{\theta_r}{\theta_s-\theta_r}\right)$,

- n is a parameter in the van Genuchten model, within the range of 0.1 to 0.3, which is suitable for most cases.

The vapor conductivity $\boldsymbol{K}_{\mathbf{vap}}(\boldsymbol{h})$ is calculated using the isothermal vapor hydraulic conductivity equation:

$$K_{\mathrm{vap}}(h) = \frac{\rho_w D}{\mu}\exp\left(\frac{h}{\lambda}\right)$$

where:

- ρ_w: Density of water (constant),

- D: Diffusion coefficient of water vapor in air (temperature-dependent),

- μ: Dynamic viscosity of water (temperature-dependent),

- λ: Characteristic length scale (empirically determined).

References: Peters (2013, 2014); Iden & Durner (2014).

3. Bimodal van Genuchten (Durner, 1994)

Description: Represents soils with two distinct pore systems (e.g., matrix and macropores).

$$\mathrm{K} = K_s \cdot \frac{(w_1 S_{e1} + w_2 S_{e2})^l \left\{ w_1 \alpha_1 \left[1 - \left(1 - S_{e1}^{n_1 - 1}\right)^{1 - \frac{1}{n_1}} \right] + w_2 \alpha_2 \left[1 - \left(1 - S_{e2}^{n_2 - 1}\right)^{1 - \frac{1}{n_2}} \right] \right\}^2}{w_1 \alpha_1 + w_2 \alpha_2}$$

where effective saturation S_{e1} and S_{e1} are defined: $S_{e1} = [\frac{1}{1+(a_1|h|)^{n_1}}]^{m_1}$, $S_{e2} = [\frac{1}{1+(a_2|\mathrm{h}|)^{n_2}}]^{m_2}$.

Reference: Durner (1994).

4. Bimodal PDI Model

Description: Combines bimodal structure with PDI decoupling (Iden & Durner, 2014; Peters, 2014).

Hydraulic Conductivity:

$$K(h) = K_s\left[\omega_1 K_{\mathrm{cap1}}(h) + \omega_2 K_{\mathrm{cap2}}(h) + (1 - \omega_1 - \omega_2) K_{\mathrm{film}}(h) + K_{\mathrm{vap}}(h)\right]$$

where:

- $K(h)$ is the hydraulic conductivity at suction h,

- K_s is the saturated hydraulic conductivity,

- ω_1 and ω_2 are the weighting factors for the first and second capillary pore systems, respectively,

- $K_{\mathrm{cap1}}(h)$ and $K_{\mathrm{cap2}}(h)$ are the capillary conductivities for the first and second pore systems, respectively,

- $K_{\mathrm{film}}(h)$ is the film conductivity,

- $K_{\mathrm{vap}}(h)$ is the vapor conductivity.

The capillary conductivities $K_{\mathrm{cap1}}(h)$ and $K_{\mathrm{cap2}}(h)$ are described by the Mualem model:

$$K_{\mathrm{cap1}}(h) = K_s\left[S_{\mathrm{cap1}}(h)\right]^s \left[\int_{h_0}^{h} \frac{1}{h'} dS_{\mathrm{cap1}}(h')\right]^2$$

$$K_{\mathrm{cap2}}(h) = K_s\left[S_{\mathrm{cap2}}(h)\right]^s \left[\int_{h_0}^{h} \frac{1}{h'} dS_{\mathrm{cap2}}(h')\right]^2$$

The film conductivity ($K_{\mathrm{film}}(h)$)is given by:

$$K_{\mathrm{film}}(h) = K_s \left(\frac{h}{h_a}\right)^{-1.5} [1 - S_{\mathrm{ad}}(h)]$$

The vapor conductivity ($K_{\text{vap}}(h)$)is calculated using the isothermal vapor hydraulic conductivity equation:

$$K_{\text{vap}}(h) = \frac{\rho_w D}{\mu} \exp\left(\frac{h}{\lambda}\right)$$

Please note that the specific forms of $S_{\text{cap}}(\text{h})$, $S_{\text{cap1}}(h)$, $S_{\text{cap2}}(h)$, and $S_{\text{ad}}(h)$ are defined in the respective papers and are not repeated here for brevity.

References: Iden & Durner (2014); Peters (2014).

References

van Genuchten, M. Th. (1980). A closed-form equation for predicting the hydraulic conductivity of unsaturated soils. Soil Science Society of America Journal, 44(5), 892–898.

Durner, W. (1994). Hydraulic conductivity estimation for soils with heterogeneous pore structure. Water Resources Research, 30(2), 211–222.

Iden, S. C., & Durner, W. (2014). Comment to “Simple consistent models for water retention and hydraulic conductivity in the complete moisture range” by A. Peters. Water Resources Research, 50, 7530–7534.

Peters, A. (2013). Simple consistent models for water retention and hydraulic conductivity in the complete moisture range. Water Resources Research, 49, 6765–6780.

Peters, A. (2014). Reply to comment by S. Iden and W. Durner on “Simple consistent models for water retention and hydraulic conductivity in the complete moisture range”. Water Resources Research, 50(9), 7535–7539.
